# Lighting a Fire: Gasdermin-Mediated Pyroptosis Remodels the Glioma Microenvironment and Promotes Immune Checkpoint Blockade Response

**DOI:** 10.3389/fimmu.2022.910490

**Published:** 2022-06-17

**Authors:** Yonghua Cai, Ke Li, Jie Lin, Xianqiu Liang, Wei Xu, Zhengming Zhan, Shuaishuai Xue, Yu Zeng, Peng Chai, Yangqi Mao, Zibin Song, Lei Han, Ye Song, Xian Zhang, Hai Wang

**Affiliations:** ^1^Department of Neurosurgery, Nanfang Hospital, Southern Medical University, Guangzhou, China; ^2^Department of Neurosurgery, Ganzhou People’s Hospital, Ganzhou, China

**Keywords:** pyroptosis, immunity, immunotherapy, gasdermins, tumor immune microenvironment, glioma

## Abstract

Pyroptosis is a proinflammatory programmed cell death pathway mediated by gasdermins. Exploring the role of pyroptosis can provide new insights into tumor malignancy. The most recent studies on pyroptosis have focused on tumor cells. However, the effects of pyroptosis on the tumor microenvironment (TME), immunotherapeutic responses, and efficacy have been neglected, especially in case of glioma. In this study, four independent glioma cohorts comprising 1,339 samples and a pan-cancer cohort comprising 10,535 tumor samples were analyzed. The relationships among pyroptosis status, prognosis, microenvironment cellular components, and clinical and biological phenotypes were investigated through the identification of pyroptosis subtypes, construction of a gasdermin-related prognostic index (GPI), and evaluation of immunological characteristics in glioma. The Genomics of Drug Sensitivity in Cancer database and “pRRophetic” package in R were used to estimate temozolomide (TMZ) sensitivity. The “Submap” package and external immunotherapy cohorts were used to investigate and confirm the role of GPI in response to and efficacy of immunotherapy in glioma. Finally, potential small-molecule compounds related to GPI were identified using the connectivity map database and mode-of-action analysis. We identified three different pyroptosis subtypes: cluster 1 (C1) characterized by a higher GPI, while cluster 2 (C2) and cluster 3 (C3) characterized by a lower GPI. The high GPI of C1 was associated with glioma progression and worse prognoses, whereas the low GPI of subtype C2 and C3 was associated with better prognoses. However, patients with high GPIs were found to be more sensitive to TMZ and immune checkpoint blockade than those with low GPIs. Furthermore, gasdermin D may be a principal potential biomarker and play key roles in pyroptosis-inducible therapy combined with immunotherapy in glioma. This study provides a clinical, biological, and molecular landscape of pyroptosis and suggests that pyroptosis of glioma cells may perform the dual function of promoting both tumorigenesis and antitumor immunity.

## Introduction

Diffuse glioma is the most common primary brain tumor, classified as World Health Organization (WHO) grades II, III, and IV (1, 2). Gliomas are highly heterogeneous tumors, ranging from low-grade glioma (LGG; WHO grade II) to high-grade glioma (HGG; WHO grades III and IV), depending on the malignancy of the tumor. Isocitrate dehydrogenase (*IDH*) mutations, chromosome arm 1p and 19q (1p/19q) codeletion, and O(6)-methylguanine-DNA methyltransferase (*MGMT*) promoter methylation are homogeneously present in gliomas (3). Patients with glioblastoma, the most malignant glioma, has a median overall survival (OS) of only 14–17 months, even when subjected to surgical resection combined with radiotherapy, temozolomide (TMZ) chemotherapy, and tumor-treating fields (4–6). Given the low survival outcomes, novel treatment strategies are urgently required to treat gliomas.

Pyroptosis, a gasdermin-mediated programmed cell death program, presents a novel paradigm for cancer treatment (7–9). The executors of pyroptosis, gasdermins, comprise a protein family encoded by six paralogous genes: gasdermin A (*GSDMA*), gasdermin B (*GSDMB*), gasdermin C (*GSDMC*), gasdermin D (*GSDMD*), gasdermin E (*GSDME*), and pejvakin (*PJVK*) (10). Gasdermins play extensive and complicated roles in cancers (11), such as esophageal and gastric tumors, non-small cell lung cancer, colorectal and breast cancers, bladder carcinoma, and melanoma (12–19). Unfortunately, only a few studies have investigated the role of pyroptosis in gliomas. A recent study showed that high GSDMD expression is associated with *IDH*-wildtype and WHO grade IV gliomas as well as shorter OS and is a response marker for TMZ treatment in glioma (20). Chen et al. reported that kaempferol, a major flavonoid present in various edible plants, increased reactive oxygen species levels and further led to GSDME-mediated pyroptosis, thereby suppressing glioma cell proliferation (20). However, most recent studies on pyroptosis have mainly focused on tumor cells, and the contingent effects of pyroptosis in the tumor immune microenvironment (TIME) have been neglected. Hence, exploring the impact of pyroptosis on the microenvironment of gliomas will provide insights into malignant progression and may even help developing novel treatment strategies, especially for immunotherapy combined with pyroptosis-inducible therapy.

In this study, to explore the effects of pyroptosis on glioma in multiple dimensions, we comprehensively analyzed the transcriptional and genetic heterogeneity of pyroptosis executors, identified three pyroptosis subtypes (C1, C2, and C3), and developed a gasdermin-related prognostic index (GPI). Our results show that the pyroptosis subtype C1 and high GPI are associated with high malignancy of glioma but may improve the sensitivity and efficacy of TMZ and immune checkpoint blockade (ICB) treatment, highlighting the value of a combination of pyroptosis-inducible therapy with chemotherapy and/or immunotherapy for glioma. Collectively, the pyroptosis of glioma cells may be a double-edged sword that promotes both tumorigenesis and antitumor immunity.

## Materials and Methods

### Data Sources and Processing

The RNA-sequencing datasets “TCGA-663”, “CGGA-325”, and “GSE43378” and the mRNA microarray dataset “CGGA-301”, along with corresponding clinical information for glioma samples, were retrieved from The Cancer Genome Atlas (TCGA) (version 28.0, https://portal.gdc.cancer.gov/), Chinese Glioma Genome Atlas (CGGA) (2021 Feb, http://www.cgga.org.cn/index.jsp) (21), and Gene Expression Omnibus databases (2021 April, https://www.ncbi.nlm.nih.gov/geo). The mRNA transcription values were converted to thousands of millions of thousand-based (TPM) values and further normalized to log2 (TPM + 1) for downstream analysis. The main study was conducted using TCGA-663 and validated using CGGA-325. The CGGA-301 and GSE43378 datasets were used to validate the GPI for glioma prognosis. The baseline clinical characteristics of the glioma samples are summarized in **Table 1**. The glioma cell line expression matrix was obtained from Cancer Cell Line Encyclopedia (CCLE) (https://portals.broadinstitute.org/ccle/about), and the unified and standardized TCGA pan-cancer dataset (n = 10535) was downloaded from the UCSC Xena database (https://xenabrowser.net/).

**Table 1:** The baseline clinical characteristics of the glioma samples.

Characteristics	TCGA-663	CGGA-325	CGGA-301	GSE43378	Total	P value
N	663	325	301	50	1339	
Age						7.200E-06
<45	319 (23.86%)	191 (14.29%)	175 (13.09%)	14 (1.05%)	699 (52.28%)	
≥45	344 (25.73%)	134 (10.02%)	124 (9.27%)	36 (2.69%)	638 (47.72%)	
Gender						2.800E-01
Female	282 (21.06%)	122 (9.11%)	121 (9.04%)	16 (1.19%)	541 (40.40%)	
Male	381 (28.45%)	203 (15.16%)	180 (13.44%)	34 (2.54%)	798 (59.60%)	
Grade						8.400E-18
II	248 (18.52%)	103 (7.69%)	117 (8.74%)	5 (0.37%)	473 (35.32%)	
III	261 (19.49%)	79 (5.90%)	57 (4.26%)	13 (0.97%)	410 (30.62%)	
IV	153 (11.43%)	139 (10.38%)	124 (9.26%)	32 (2.39%)	448 (33.46%)	
NA	1 (0.07%)	4 (0.30%)	3 (0.22%)	0 (0.0e+0%)	8 (0.60%)	
status						2.100E-29
Alive	415 (30.99%)	96 (7.17%)	112 (8.36%)	8 (0.60%)	631 (47.12%)	
Dead	247 (18.45%)	220 (16.43%)	187 (13.97%)	42 (3.14%)	696 (51.98%)	
NA	1 (0.07%)	9 (0.67%)	2 (0.15%)	0 (0.0e+0%)	12 (0.90%)	
OS						
Mean±SD	2.29±2.44	3.98±4.03	4.32±4.08	2.16±1.78	3.14±3.40	
Median [min-max]	1.55 [0.0E+0,17.60]	1.93 [0.05,13.18]	2.23 [0.06,13.22]	1.49 [0.05,8.27]	1.67 [0.0E+0,17.60]	

N, the number of glioma samples; NA, unknown; OS, overall survival.

### Online Databases and Tools

The cBioPortal (http://www.cbioportal.org) (22) was used to retrieve and visualize mutations and copy-number alterations (CNA) of gasdermins in the “Merged Cohort of LGG and GBM (TCGA, Cell 2016).” The Human Protein Atlas database (version 21.0, https://www.proteinatlas.org/) was used to explore the protein expression levels of gasdermins. STRING (https://string-db.org/) (23) was used to identify gasdermin-related molecules, and Gene Ontology (GO) and Kyoto Encyclopedia of Genes and Genomes (KEGG) were used for term enrichment.

### Identification of Pyroptosis Subtypes

Unsupervised cluster analysis was performed to identify the pyroptosis-related subtypes in glioma using the “ConsensusClusterPlus” R package (24), based on the expression of pyroptosis-related molecules, using agglomerative pam clustering with a 1-Pearson correlation distance and resampling 80% of the samples for 10 repetitions. The optimal number of clusters was determined using an empirical cumulative distribution function plot. Mutation data were downloaded from TCGA and visualized using the “maftools” R package (25) for identifying the somatic mutation landscape in distinct pyroptosis-related subtypes.

### GPI and Nomogram Construction

We used the “glmnet” R package (26) to integrate survival time, survival status, and gene expression data for regression analysis using the Lasso-Cox method. In addition, we set up a 10-fold cross-validation to obtain the optimal model to yield the GPI equation with the coefficient multiplied by mRNA expression. The coefficient was derived by running “glmnet” on the entire TCGA-663 dataset with the optimal lambda value. A nomogram was created to predict the probability of OS based on the GPI combined with clinical characteristics through the “rms” R package and evaluated using a calibration plot, which compares nomogram-predicted probability with observed survival probability. Decision curve analysis (DCA) was used to evaluate the clinical application of the nomogram by assessing the net benefits of the prediction model at different threshold probabilities and concordance indices.

### Gene Set Enrichment Analysis

We obtained the GSEA software (version 3.0) from the GSEA website (http://software.broadinstitute.org/gsea/index.jsp) (27) and downloaded the “h.all.v7.4.symbols.gmt” subset from the Molecular Signature Database (http://www.gseamsigdb.org/gsea/downloads.jsp) (28) to explore GPI-related pathways and molecular mechanisms based on gene expression profiles and GPI groups (high and low, separated by the median value). We adjusted the minimum gene set to 5 and the maximum gene set to 5000 and performed 1000 resamplings. P-value < 0.05 and FDR < 0.25 were considered statistically significant. Finally, the GSEA results were visualized using the “ggplot2” R package.

### Evaluation of Immunological Characteristics

The “estimate” R package (29) was used to calculate the immune score and stromal score for each glioma sample. The “immunedeconv” R package (30) was utilized to estimate TME infiltrating cells for each glioma sample. The tumor mutation burden (TMB) score of each sample was calculated using the “tmb” function of the “maftools” R package (25). The microsatellite instability (MSI) score for each sample was obtained from a previous study (31). The stemness indices (mRNA expression-based stemness index, mRNAsi) for each sample were calculated using the OCLR algorithm developed by Malta et al. (32). The “deconvo_IPS” method of the “IOBR” R package (33) was used to assess the antigen processing cell (MHC), effector cell (EC), suppressor cell (SC), and checkpoint (CP) scores and immunophenoscore (IPS) of each tumor sample.

### Correlation of GPI With TMZ Sensitivity

The TMZ sensitivity of each sample was estimated using Genomics of Drug Sensitivity in Cancer (GDSC, https://www.cancerrxgene.org/) (34), which is the largest publicly available pharmacogenomics database. The estimated half-maximal inhibitory concentration (IC_50_) was calculated using ridge regression, and the prediction accuracy was determined using the “pRRophetic” R package (35). All parameters were set to default values with the removal of the batch effect of “combat” and “allSoldTumours” tissue types, and duplicate gene expression was summarized as the mean value.

### Correlation of GPI With ICB Response

The potential ICB response was predicted using Submap (36)—a tool for comparing expression profiles—in GenePattern (https://cloud.genepattern.org/gp). We used the Submap algorithm combined with human immunotherapy transcriptome data from Roh et al. (37) to further investigate the predictive value of GPI in anti-PD1 and anti-CTLA4 immunotherapy response. Furthermore, several immunotherapy cohorts from Snyder et al. (38), Nathanson et al. (39), Mariathasan et al. (40), and Rose et al. (41) were used to validate the predictive value of GPI in the response to and efficacy of immunotherapy.

### Candidate Small-Molecule Drugs Based on GPI

First, weighted co-expression gene modules identified and the module-trait relationships was determined using the “WGCNA” R package (42). The module with the |correlation coefficient| > 0.5 and P-value < 0.05 was considered as a meaningful module in this study. Second, only one module associated with immunity was identified using the STRING database (https://string-db.org/) (42). Then, differentially expressed genes (DEGs) were identified between the high- and low-GPI groups using the “limma” R package. Genes with P < 0.05 and |FC| > 1 were considered significant DEGs. The GO functional and KEGG pathway enrichment analyses of GPI- and immune-related DEGs were then performed using the “clusterProfiler” R package (43). Based on the upregulated and downregulated DEGs, candidate small-molecule drugs and mechanisms of action were predicted using the connectivity map (CMap, http://portals.broadinstitute.org/cmap/) database and CMap mode-of-action (MOA) analysis (44).

### Statistical Analysis

All statistical analyses were conducted using R software (version 4.0.2), with a P-value < 0.05 (two-tailed) indicating significant differences. Unpaired *t*-tests were performed to compare two normally distributed variables. The Wilcoxon rank-sum test was performed to compare two non-normally distributed variables. The Kruskal–Wallis test (nonparametric method) or one-way analysis of variance (parametric method) was used for comparisons of three or more variables. Pearson and Spearman correlation coefficients were used to determine correlations between variables. The “survfit” function in the “Survminer” R package was used to evaluate prognostic differences between the two groups. Kaplan–Meier (KM) analysis was used to generate survival curves, and the log-rank test was performed to determine statistically significant differences. The receiver operating characteristic (ROC) curve was used to assess the prognosis prediction performance, and the area under the curve (AUC) was calculated using the “timeROC” R package.

## Results

### Aberrant Expression, Genetic Alteration, and Prognostic Value of Gasdermins in Glioma

**Figure 1:**
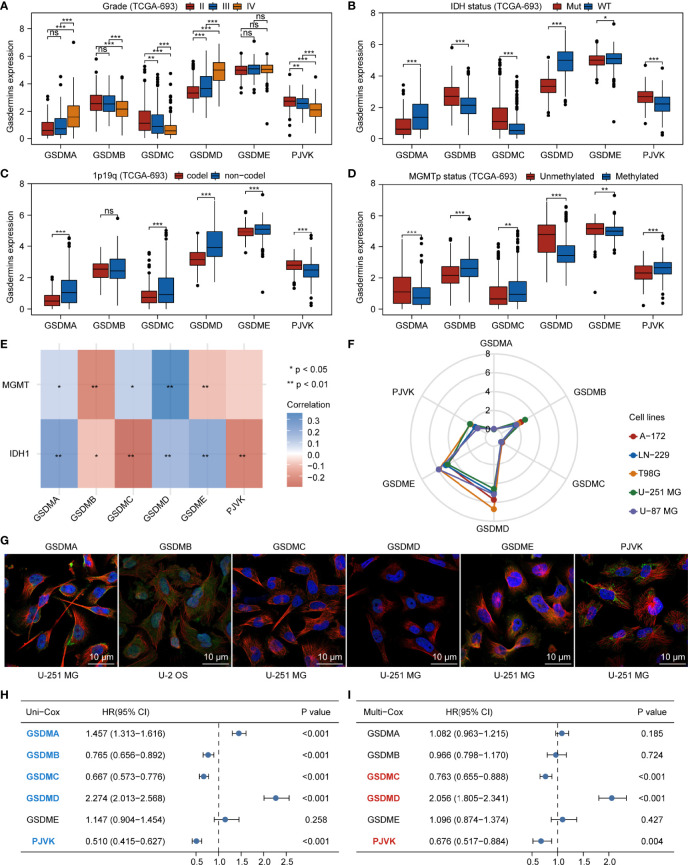
Aberrant expression and prognostic value of gasdermins in glioma. Boxplots showing comparison of gasdermin expression in different grades **(A)**, *IDH* mutation statuses **(B)**, 1p19q codeletion statuses **(C)**, and *MGMT*-promoter methylation statuses **(D)** of glioma samples. **(E)** Heatmap showing correlation between gasdermin expression and *IDH1* or *MGMT* expression. **(F)** Radar plot showing gasdermin expression in different glioma cell lines. **(G)** The results of immunofluorescence from HPA database showing the subcellular distribution of the gasdermin protein. **(H, I)** Forest plots showing the results of univariate and multivariate Cox regression analyses for gasdermins. *P < 0.05; **P < 0.01; ***P < 0.001; ns, not significant.

We comprehensively analyzed the molecular characteristics and prognostic significance of gasdermins—the executors of pyroptosis. The analysis of the data of glioma samples from TCGA-693 showed that GSDMA and GSDMD were more highly expressed in grade IV than in grade II–III samples. Furthermore, GSDMB, GSDMC, and PJVK expression was lower in grade IV than in grade II–III samples, while GSDME expression showed no significant differences among grades (**Figure 1A**). As shown in **Figure 1B**, GSDMA, GSDMD, and GSDME expression was higher in *IDH*-wildtype than in *IDH*-mutant samples, while GSDMB, GSDMC, and PJVK expression was lower in *IDH*-wildtype than in mutant samples. GSDMA, GSDMC, GSDMD, and GSDME expression was higher in 1p19q non-codel than in codel samples (**Figure 1C**), while PJVK expression was lower in 1p19q non-codel than in codel samples. Additionally, GSDMB expression showed no significant differences between the different 1p19q statuses. As shown in **Figure 1D**, GSDMA, GSDMD, and GSDME expression was lower in the *MGMT*-promoter methylated samples than in the unmethylated ones, while GSDMB, GSDMC, and PJVK expression was higher in *MGMT*-promoter methylated samples than in the unmethylated ones. Similar findings were observed for the CGGA-325 cohort (**Figures S1A–D**). **Figure 1E** shows that *IDH1* expression was positively related to GSDMA, GSDMD, and GSDME expression but negatively related to GSDMB, GSDMC, and PJVK expression. However, MGMT expression was positively related to GSDMA, GSDMC, and GSDMD expression but negatively related to GSDMB and GSDME expression. The analysis of glioma cell lines from CCLE showed that GSDMD and GSDME were more highly expressed in all glioma cell lines (including A-172, LN-229, T98G, U-251 MG, and U-87 MG) than GSDMB and PJVK, but GSDMA and GSDMC were poorly expressed in those glioma cell lines (**Figure 1F**). To explore the subcellular distribution of gasdermin expression in U-251 MG cells, we analyzed the results of immunofluorescence (ICC-IF) and confocal microscopy from the HPA database, and found that GSDMA was mainly located in the nucleoplasm, plasma membrane, and cytosol and GSDMB in the nucleoplasm and cytosol. As HPA did not contain U-251 MG-related ICC-IF results of GSDMB, we acquired the ICC-IF data of the U-2 OS cell line. We found that GSDMD was primarily located in the nucleoplasm, GSDME in the cytosol, and GSDMC and PJVK in the mitochondria (**Figure 1G**). To analyze the genetic characteristics of gasdermins, we explored the genetic alterations in 794 glioma samples with mutation and CNA data on the cBioPortal database. The results showed that gasdermins were altered in 48 (6%) of the 794 samples, but no gasdermin showed alternations of more than 3% (**Figure S1E**). Univariate and multivariate Cox regression analyses were performed to analyze the prognostic value of gasdermins in gliomas. The univariate Cox survival analysis indicated that GSDMA (P < 0.001), GSDMB (P < 0.001), GSDMC (P < 0.001), GSDMD (P < 0.001), and PJVK (P < 0.001) expression was strongly associated with clinical outcomes, but GSDME expression (P = 0.258) had no significant effect on survival **(** **Figure 1H**). Multivariate Cox survival analyses showed that GSDMC (P < 0.001), GSDMD (P < 0.001), and PJVK (P = 0.004) were independent prognostic factors for gliomas (**Figure 1I**). The immunohistochemistry (IHC) results from the HPA database showed that GSDMC staining was not detected in normal brain, LGG, and HGG tissues, whereas GSDMD staining was not detected in normal brain tissues, was low in LGG tissues, and was medium in HGG tissues (**Figure S1F**). IHC staining data for PJVK were not available in the HPA database and therefore could not be assessed along these lines. These results suggest that gasdermins have potential target-treatment value for glioma.

### Gasdermins are Correlated With Immune Checkpoints and Glioma Stem Cell, Glioma-Associated Stromal Cell, and Glioma-Associated Immune Cell Biomarkers

**Figure 2:**
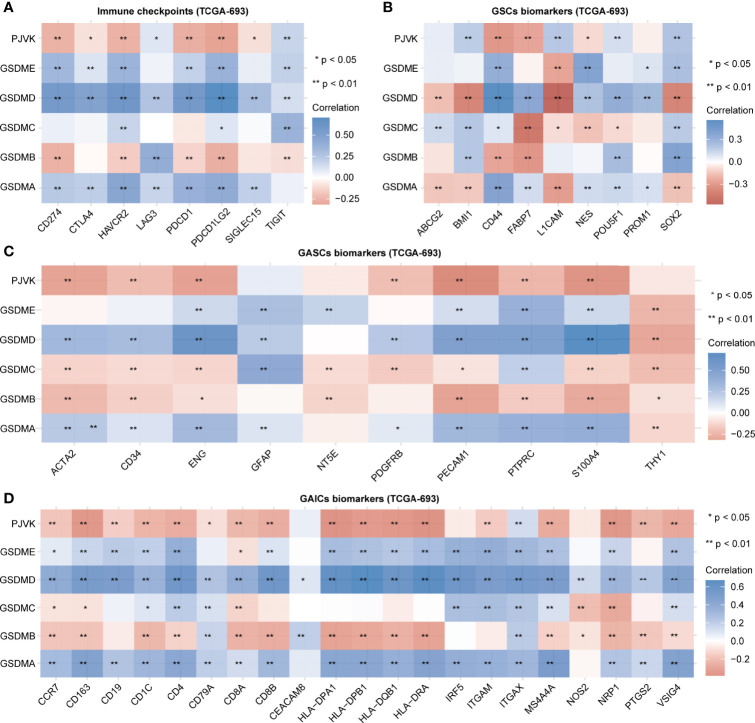
Gasdermins are correlated with immune checkpoints and biomarkers of GSCs, GASCs, and GAICs. Heatmaps depicting correlation between gasdermin expression and immune checkpoints **(A)**, GSC markers **(B)**, GASC markers **(C)**, and GAIC biomarkers **(D)**. *P < 0.05; **P < 0.01; ns, not significant.

Initially, we explored the potential association between gasdermins and the major components of the glioma microenvironment. We extracted the transcript and expression values of eight immune checkpoints (CD274, CTLA4, HAVCR2, LAG3, PDCD1, PDCD1LG2, SIGLEC15, and TIGIT). **Figure 2A** shows that the expression of GSDMA, GSDMD, and GSDME was positively correlated with that of most immune checkpoints, while the expression of GSDMB and PJVK was negatively correlated with that of most immune checkpoints. The specific statistical data on the correlation between gasdermins and immune checkpoints are presented in **Table S1**. We summarized the data on nine glioma stem cell (GSC) biomarkers (ABCG2, BMI1, CD44, FABP7, L1CAM, NES, POU5F1, PROM1, and SOX2) from published studies (45–47). **Figure 2B** shows that all gasdermins were positively correlated with most GSC biomarkers; GSDMD was highly associated with all GSC markers. The specific statistical data on the correlation between gasdermins and GSC biomarkers are presented in **Table S2**. Next, we summarized the data on 10 glioma-associated stromal cell (GASC) biomarkers (ACTA2, CD34, ENG, GFAP, NT5E, PDGFRB, PECAM1, PTPRC, S100A4, and THY1) from a published study (48). **Figure 2C** shows that the expression of GSDMA, GSDMD, and GSDME was positively correlated with those of most GASC biomarkers, while those of GSDMB, GSDMC, and PJVK are negatively correlated. The specific statistical data for the correlation between gasdermins and GASC biomarkers are presented in **Table S3**. Finally, we summarized the data on 21 glioma-associated immune cell (GAIC) biomarkers (CCR7, CD163, CD19, CD1C, CD4, CD79A, CD8A, CD8B, CEACAM8, HLA-DPA1, HLA-DPB1, HLA-DQB1, HLA-DRA, IRF5, ITGAM, ITGAX, MS4A4A, NOS2, NRP1, PTGS2, and VSIG4) from existing studies (49, 50). **Figure 2D** shows that GSDMA, GSDMD, and GSDME expression was positively correlated with that of most GAIC biomarkers, while GSDMB and PJVK expression was negatively correlated. The specific statistical data for the correlation between gasdermins and GAIC biomarkers are presented in **Table S4**. The CGGA-325 dataset showed similar results (**Figures S2A–D**). These results suggest that gasdermin-mediated pyroptosis has potential implications in tumor cell heterogeneity and the glioma immune microenvironment.

### Three Glioma Pyroptosis Subtypes With Distinct TIME Features Identified *via* Gasdermin-Related Genes

We selected 54 gasdermin-related genes from the STRING database (**Figure S3A** and **Table S5**), most of which were pyroptosis-related molecules (9, 51, 52). GO functional enrichment analysis showed that the gasdermin-related genes were not only enriched in pyroptosis (GO:0070269) but also in positive regulation of T cell cytokine production (GO:0002726), cytokine production involved in the immune response (GO:0002367), positive regulation of interleukin-1 beta secretion (GO:0050718), regulation of T-helper 1 type immune response (GO:0002825), positive regulation of T-helper 1 cell cytokine production (GO:2000556), interleukin-18-mediated signaling pathway (GO:0035655), and positive regulation of T-helper 2 cell differentiation (GO:0045630) (**Figure S3B** and **Table S6**). These results imply that gasdermin-related genes are associated with the pyroptosis signaling pathway as well as with other immune system processes.

**Figure 3:**
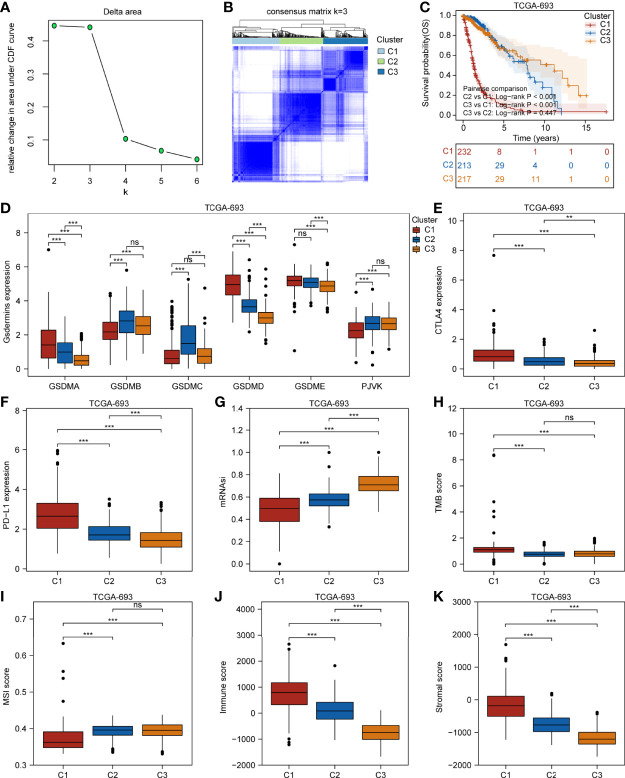
Three glioma pyroptosis subtypes based on the expression of gasdermin-related genes. **(A)** Relative change in area under CDF curve for k = 2 to 6. **(B)** Consensus clustering matrix for k = 3. **(C)** Survival analysis of the three pyroptosis subtypes C1–C3. Boxplots showing comparison of the gasdermin expression **(D)**, CTLA4 expression **(E)**, PD-L1 expression **(F)**, mRNAsi **(G)**, TMB score **(H)**, MSI score **(I)**, immune score **(J)**, and stromal score **(K)** in the three pyroptosis subtypes. **P < 0.01; ***P < 0.001; ns, not significant.

We further applied a consensus clustering method based on the expression profiles of the pyroptosis-related molecules and found that the optimal cluster number of glioma samples was three (K = 3) (**Figure 3A**). The division of the glioma samples of TCGA-693 into three pyroptosis subtypes (C1, C2, and C3) is shown in **Figure 3B**, and an overview of the pyroptosis-related molecule expression landscape in TCGA-693 is shown in **Figure S4A**. The glioma samples in C2 or C3 had better clinical outcomes than those in C1 (**Figure 3C**). **Figure 3D** shows the expression levels of aberrant gasdermins in different pyroptosis subtypes. GSDMA, GSDMD, and GSDME expression was higher in C1 than in C2 and C3; GSDMB expression was higher in C2 than in C1 and C3; and PJVK was expressed at a lower level in C1 than in C2 and C3. CGGA-325 showed similar results (**Figures S4B–E**). These results suggest that the three pyroptosis subtypes represent three major and different pyroptosis statuses with distinct OS in glioma.

**Figure 4:**
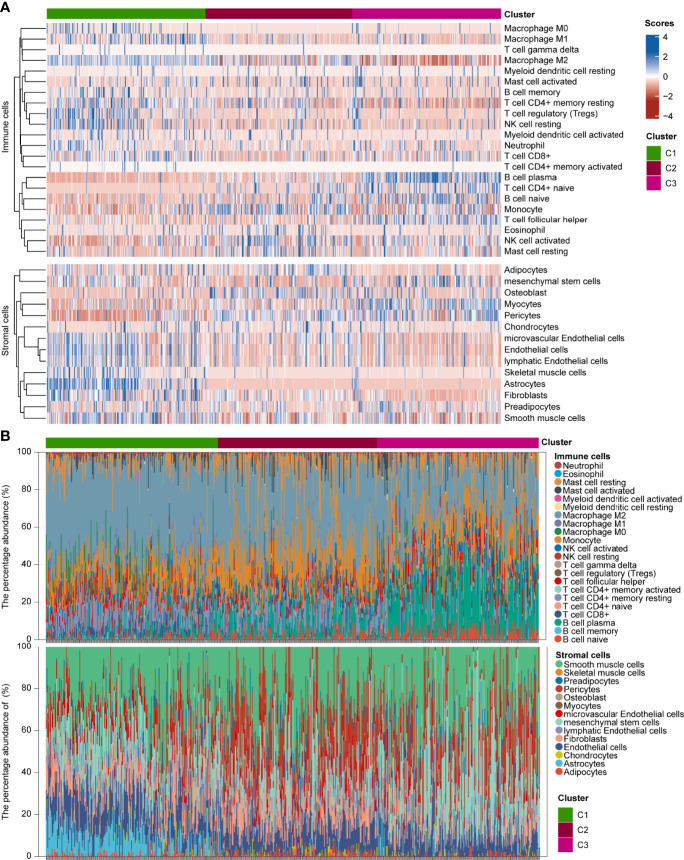
Infiltration estimations of GAICs and GASCs in three glioma pyroptosis subtypes. **(A)** Heatmap depicting proportion and subtypes of infiltrating GAICs and GASCs in the three pyroptosis subtypes. **(B)** Stacked plot of the percentage abundance of infiltrating GAICs and GASCs for each sample.

To explore the genetic alterations in different pyroptosis subtypes, we analyzed the top 10 mutated genes in TCGA glioma samples. As shown in **Figures S5A–C**, 191 samples had mutations with a frequency of 83.41% in C1, 194 samples with 93.72% in C2, and 202 samples with 94.39% in C3. Missense mutations were the most common in all three clusters. TP53 had the highest mutation frequency (31%), followed by *EGFR* (27%), *TTN* (25%), and *PTEN* (25%) in C1; *IDH1* had the highest mutation frequency (89%), followed by *TP53* (67%) and *ATRX* (49%) in C2; and *IDH1* had the highest mutation frequency (82%), followed by *CIC* (34%) and *TP53* (31%) in C3. These results imply that genetic features may influence the pyroptosis status in gliomas. To analyze the TIME characteristics of different pyroptosis subtypes, we compared PD-L1 expression, CTLA4 expression, mRNAsi score, TMB score, MSI score, immune score, stromal score, and infiltrating cells in the three glioma sample clusters. **Figures 3E, F** and **Figures S5D, E** show that PD-L1 and CTLA4 expression was higher in C1 than in C2 or C3. The mRNAsi score was higher for C3 than for C1 or C2 (**Figure 3G**). The TMB score was higher for C1 than for C2 or C3, while the MSI score was lower in C1 than in C2 or C3 (**Figures 3H, I**). **Figures 3G–K** and **Figures S5F, G** show that the immune and stromal scores were higher for C1 than for C2 and C3. Distinct proportions and subtypes of infiltrating immune and stromal cells existed between C1 and C3, while C2 likely had an intermediate state between the two (**Figure 4A** and **Figures S6A, B**). The percentage abundance of M2 macrophages, M1 macrophages, CD8^+^ T cells, astrocytes, and endothelial cells in C1 were significantly higher in C1 than those in C2 and C3, while the percentage abundance of plasma B cells and mesenchymal stem cells was lower in C1 than in C2 and C3 (**Figure 4B**). These results imply that different pyroptosis statuses may promote or suppress the formation of an immunosuppressive TME, further influencing the progression and prognosis of glioma.

### Development and Validation of GPI for Glioma

**Figure 5:**
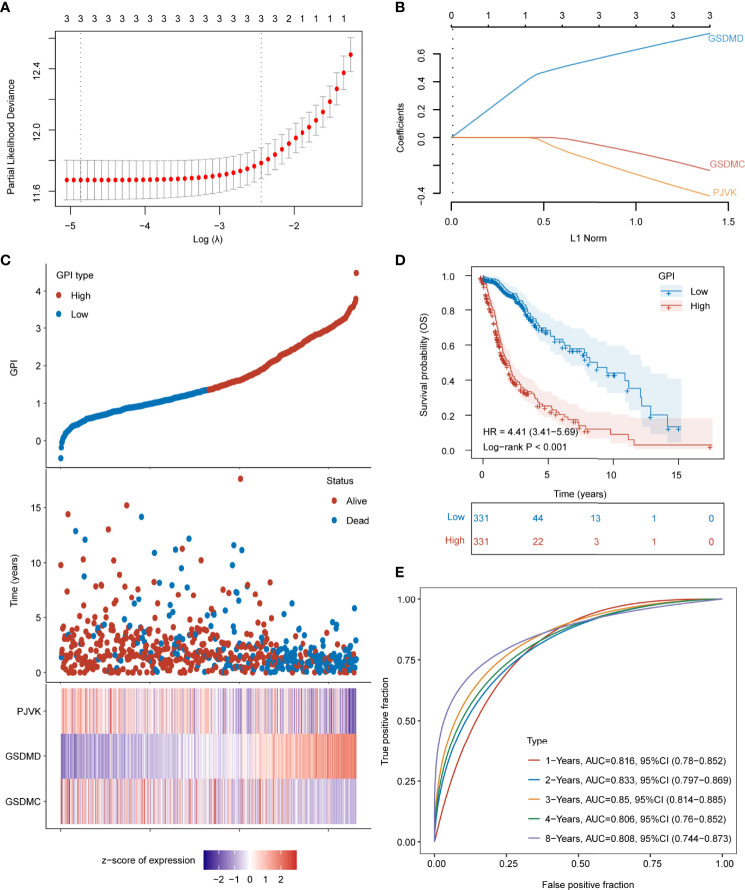
Development of GPI for glioma. **(A)** Partial likelihood deviance versus log (λ), where λ is the tuning parameter. **(B)** The coefficients of GSDMC, GSDMD, and PJVK are shown by the λ parameter. The abscissa represents the λ value, and ordinate represents the coefficients of the corresponding independent variable. (**C**, top) Scatterplot showing GPI from low to high; (**C**, middle) scatter plot distribution represents survival time and survival status of different samples with corresponding GPI; (**C**, bottom) heatmap showing GSDMC, GSDMD, and PJVK expression from GPI signature. **(D)** Survival analysis between GPI-high and GPI-low groups. **(E)** ROC curve of GPI for OS.

Further, we developed GPI based on the gasdermins expression matrix using the Lasso-Cox method in TCGA-693 training set. **Figure 5A** shows the partial likelihood deviance versus log (λ), where λ is the tuning parameter. **Figure 5B** shows the optimal lambda (lambda.min = 0.0077) and the corresponding coefficients of the selected factors (GSDMC = -0.2321, GSDMD = 0.7436, PJVK = -0.4135). The formula for the final scoring model is as follows:


$$\begin{array}{ccc} GPI=\left(-0.2321\times GSDMC\;expression\right) & +\left(0.7436\times GSDMD\;expression\right) & +\left(-0.4135\times PJVK\;expression\right) \end{array}$$

**Figure 6:**
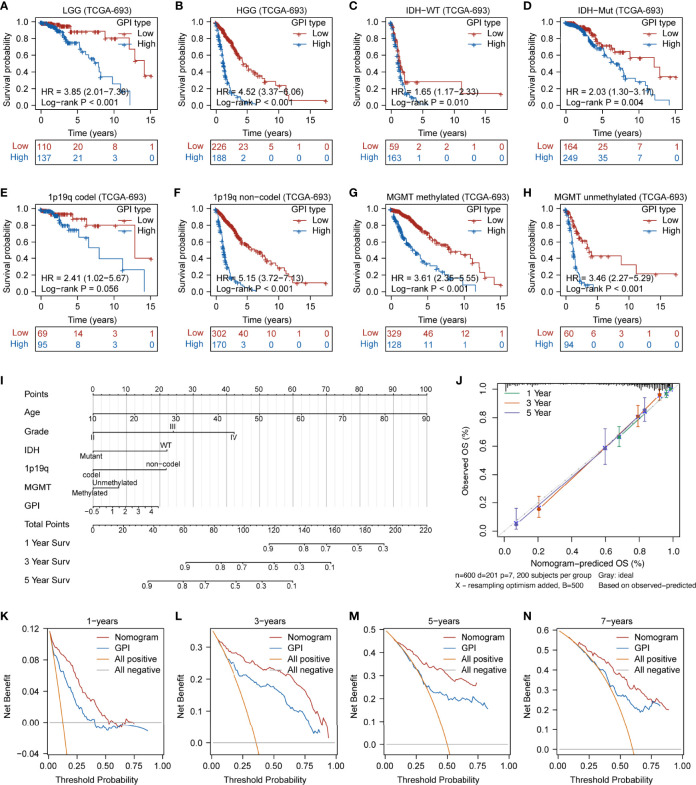
Prediction accuracy of GPI in glioma samples with different clinical characteristics and prognostic nomogram construction. Survival analysis between GPI-high and GPI-low in LGG **(A)**, HGG **(B)**, *IDH*-WT **(C)**, *IDH*-Mut **(D)**, 1p19q-codel **(E)**, 1p19q non-codel **(F)**, *MGMT*-promoter methylated **(G)**, and *MGMT*-promoter unmethylated **(H)** groups in glioma. **(I)** Nomogram was developed with the age, grade, *IDH* mutational status, 1p19q codeletion status, *MGMT*-promoter methylation status, and GPI. **(J)** Calibration plot for nomogram. **(K–N)** Decision curve analysis (DCA) of 1-, 3-, 5-, and 7-year overall survival for GPI and nomogram.

The dotted line represents the GPI ranging from low to high and divides the patients into low- and high-GPI groups (GPI-L and GPI-H, respectively) based on the median value (**Figure 5C****, upper**). The alive-status samples were mainly distributed in the GPI-L group, whereas the dead-status samples in the GPI-H group (**Figure 5C****, middle**). The heatmap of the expression profiles of the prognostic genes shows that GSDMC and PJVK were highly expressed in the GPI-L group, whereas GSDMD was highly expressed in the GPI-H group (**Figure 5C****, lower**). **Figure 5D** shows that patients with higher GPIs had significantly worse prognoses than those with low GPI (log-rank P < 0.001). The time-dependent ROC curve (**Figure 5E**) shows that GPI has a strong prognostic value for glioma and can help predict both short-term and long-term survival (1-year AUC = 0.816; 2-year AUC = 0.833; 3-year AUC = 0.850; 4-year AUC = 0.806; 8-year AUC = 0.808). Three independent glioma cohorts (“CGGA-325”, “CGGA-301”, and “GSE43378”) were used as validation sets to verify the predictive power of GPI. **Figures S7A–C** show that a high GPI significantly correlates with a worse prognosis in all three validation sets. The results in **Figures S7D–F** verify that the predictive accuracy of GPI is high in all independent validation sets (“CGGA-325,” 1-year AUC = 0.683, 3-year AUC = 0.754, 5-year AUC = 0.786; “CGGA301,” 1-year AUC = 0.601, 3-year AUC = 0.633, 5-year AUC = 0.625; “GSE43378,” 1-year AUC = 0.665, 3-year AUC = 0.836, 5-year AUC = 0.700); these results are consistent with those of TCGA-693 training set. In addition, we verified the prediction stability of GPI for gliomas in different clinical or molecular subgroups. **Figures 6A–H** show that the higher GPIs commonly correlated with shorter survival time, regardless of the LGG (log-rank P < 0.001), HGG (log-rank P < 0.001), *IDH*-wildtype (log-rank P = 0.01), *IDH*-mutant (log-rank P = 0.004), 1p19q codel (log-rank P = 0.056), 1p19q non-codel (log-rank P < 0.001), *MGMT*-promoter methylated (log-rank P < 0.001), and *MGMT*-promoter unmethylated (log-rank P < 0.001) groups. Similar results were observed for the CGGA-325 cohort (**Figures S7G–N**). These results demonstrate that GPI is generally stable and accurate for the prognosis of glioma.

To further refine and optimize the prediction performance of GPI for glioma, we integrated GPI and clinical factors (including age, grade, *IDH* mutational status, 1p19q codel status, and *MGMT*-promoter methylation status) to construct an OS nomogram model (**Figure 6I**). **Figure 6J** shows that the nomogram calibration curves of 1-year (green line), 2-year (red line), and 5-year (purple line) OS are close to the ideal curve (dashed diagonal line), indicating that there is a good agreement between predicted and observed probabilities. Finally, we performed DCA to evaluate the clinical utility of GPI. **Figures 6K–N** show that the updated nomogram model integrating GPI and clinical characteristics provided a greater predictive net benefit than a single GPI for a wide range of decision thresholds, including both short-term and long-term survival.

### GPI Is Associated With Clinical Features and Immunity of Glioma Patients

**Figure 7:**
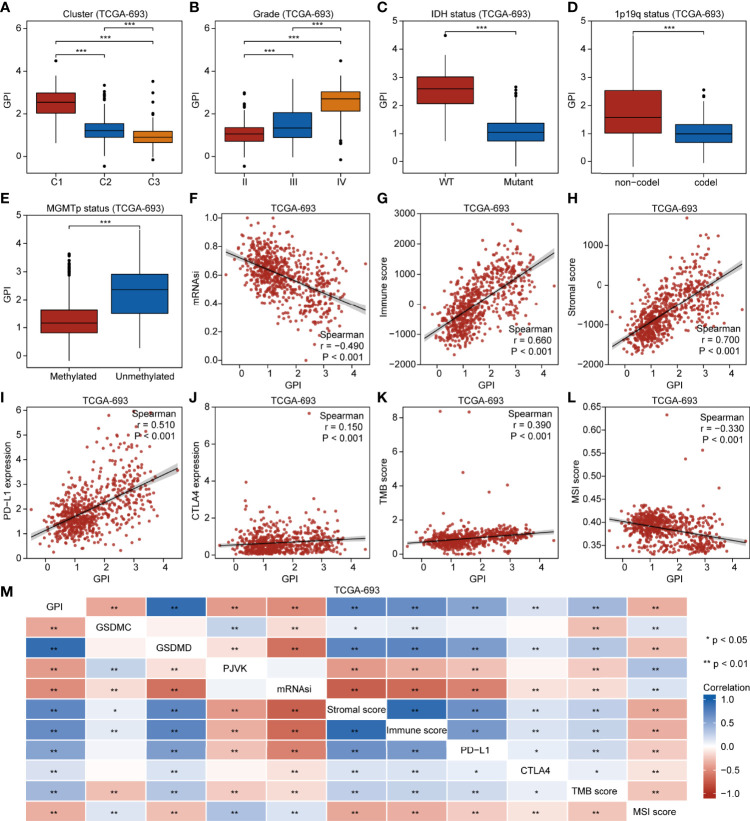
GPI is correlated with clinical characteristics, molecular features, and immunity in glioma. Boxplots showing comparison of GPI in different pyroptosis subtypes **(A),** grades **(B)**, *IDH* mutation statuses **(C)**, 1p19q codeletion statuses **(D)**, and *MGMT*-promoter methylation statuses **(E)**. Scatterplots showing correlations of GPI with mRNAsi **(F)**, immune score **(G)**, stromal score **(H)**, *PD-L1* expression **(I)**, *CTLA4* expression **(J)**, TMB score **(K)**, and MSI score **(L)**. **(M)** Heatmap indicating correlation between GPI, *GSDMC* expression, *GSDMD* expression, *PJVK* expression, mRNAsi, immune score, stromal score, *PD-L1* expression, *CTLA4* expression, TMB score, and MSI score. *P < 0.05; **P < 0.01; ns, not significant.

**Figure 8:**
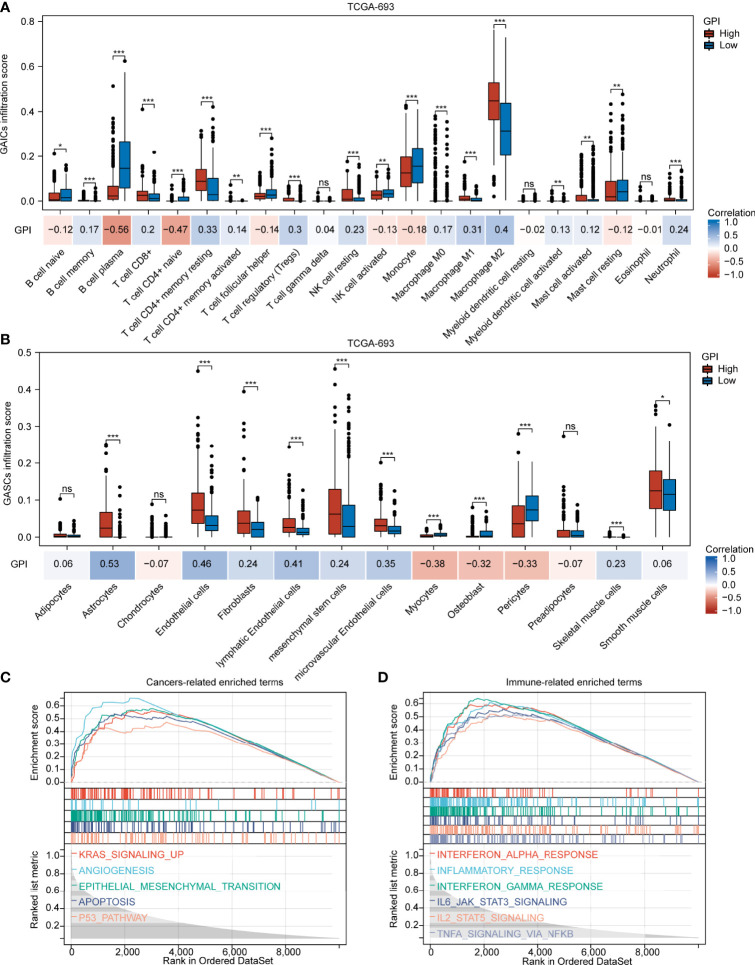
GPI is highly associated with GAIC and GASC infiltration. Boxplots showing comparison of the GAIC infiltration **(A)** and GASC infiltration **(B)** between the GPI-high and GPI-low groups. **(C, D)** Enrichment plots from GSEA. *P < 0.05; **P < 0.01; ***P < 0.001; ns, not significant.

Our findings demonstrate that different pyroptosis subtypes may exhibit different pyroptosis statuses. **Figure 7A** shows that the pyroptosis subtype C1 has the highest GPI, while the pyroptosis subtype C3 has the lowest GPI. These results indicate that, to some extent, GPI can be used as a quantitative attribute of the intrinsic pyroptosis status of glioma. To further explore the function of pyroptosis, we analyzed the correlation between GPI and different clinical features, molecular characteristics, and immune-related indices. **Figure 7B** shows that GPI progressively increases with the glioma grade. **Figures 7C–E** show that samples with *IDH*-wildtype, 1p19q non-codel, and *MGMT*-promoter unmethylated status had higher GPIs than those with *IDH*-Mut, 1p19q-codel, and *MGMT*-promoter methylated status. **Figures 7F–L** show that GPI was negatively correlated with mRNAsi (r = -0.490, P < 0.001) and MSI score (r = -0.330, P < 0.001) and positively correlated with immune score (r = 0.660, P < 0.001), stromal score (r = 0.700, P < 0.001), PD-L1 expression (r = 0.510, P < 0.001), CTLA4 expression (r = 0.150, P < 0.001), and TMB score (r = 0.390, P < 0.001). In addition, we analyzed the correlation between gasdermins (GSDMC, GSDMD, and PJVK) and GPI, mRNAsi, stromal score, immune score, PD-L1 and CTLA4 expression, TMB, and MSI. The results are shown in the form of a correlation heatmap (**Figure 7M**), and the specific statistical data are summarized in **Table S7**. Similar findings were obtained from the CGGA-325 dataset (**Figures S8A–I**). To further determine which subsets of infiltrating cells are mainly affected by pyroptosis, we compared the infiltration degrees of immune and stromal cells between the GPI-H and GPI-L groups and calculated the correlation coefficients of GPI with the proportion of specific infiltrating cells. **The upper panel** of **Figure 8A** shows that the infiltration of memory B cells, CD8^+^ T cells, resting memory CD4^+^ T cells, activated memory CD4^+^ T cells, regulatory T cells, resting NK cells, M0 macrophages, M1 macrophages, M2 macrophages, activated myeloid dendritic cells, activated mast cells, and neutrophils was higher in the GPI-H group than in the GPI-L group, while that of naïve B cells, plasma B cells, naïve CD4^+^ T cells, follicular helper T cells, activated NK cells, monocytes, and resting mast cells was lower. **The lower panel** of **Figure 8A** shows strong positive and negative correlations between GPI and each immune cell subtype, especially plasma B cells (r = -0.56), naïve CD4^+^ T cells (r = -0.47), resting memory CD4^+^ T cells (r = 0.33), regulatory T cells (r = 0.30), M1 macrophages (r = 0.31), and M2 macrophages (r = 0.40). **The upper panel** of **Figure 8B** shows that the infiltration of astrocytes, endothelial cells, fibroblasts, lymphatic endothelial cells, mesenchymal stem cells, microvascular endothelial cells, skeletal muscle cells, and smooth muscle cells was higher in the GPI-H group than in the GPI-L group, while that of myocytes, osteoblasts, and pericytes was lower. **The lower panel** of **Figure 8B** shows strong positive and negative correlations between GPI and each stromal cell subtype, especially astrocytes (r = 0.53), endothelial cells (r = 0.46), lymphatic endothelial cells (r = 0.41), microvascular endothelial cells (r = 0.35), myocytes (r = -0.38), osteoblasts (r = -0.32), and pericytes (r = -0.33). Similar results were obtained from the CGGA-325 dataset (**Figures S9A, B**). These results suggest that pyroptosis, as an immunogenic cell death mechanism, results in an imbalance in TIME by altering the proportion of immune cell and stromal cell infiltration.

To further explore the biological processes and pathways influenced by pyroptosis in gliomas, we performed GSEA. **Figure 8C** shows that several common cancer-related signaling pathways, including *KRAS* signaling up, angiogenesis, epithelial mesenchymal transition, apoptosis, and the *P53* pathway, were active in the GPI-H group. **Figure 8D** shows significant activation of many immune-related signaling pathways in the GPI-H group, including interferon alpha response, inflammatory response, interferon gamma response, *IL6/JAK/STAT3* signaling, *IL2/STAT5* signaling, and TNFA signaling *via NFκB*. However, GSEA did not identify any significantly enriched pathways in the GPI-L group. All GSEA results are presented in **Table S8**. These results imply that these cancer- and immune-related pathways may be involved in the regulation of pyroptosis and TIME balance in glioma.

### Patients With High GPIs Are More Sensitive to TMZ and Anti-PD1 Therapy

**Figure 9:**
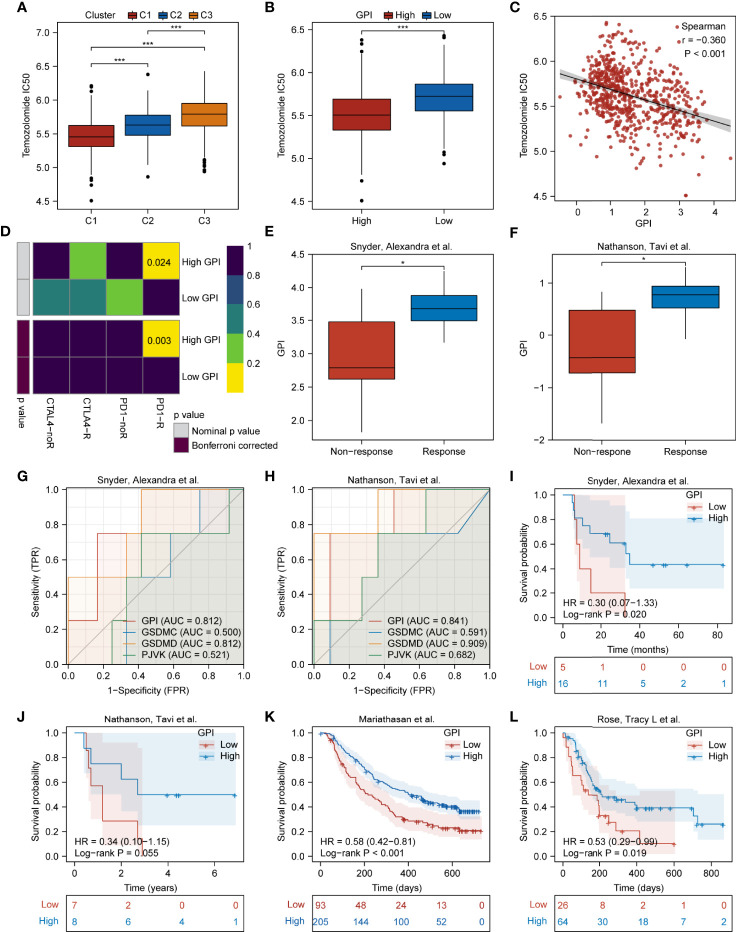
Patients with high GPIs are more sensitive to TMZ and ICB therapy. Boxplots showing comparison of the TMZ IC_50_ among the three pyroptosis subtypes **(A)** and between GPI-high and GPI-low groups **(B)**. Scatterplots showing the correlation of TMZ IC_50_ with GPI **(C)**. **(D)** Submap analysis showing differences in sensitivity of GPI-high and GPI-low groups to anti-PD1 and anti-CTLA4 immunotherapy. **(E, F)** Boxplots showing comparison of the GPI level between responders and non-responders. **(G, H)** ROC curve of GPI for immunotherapy response. **(I–L)** Survival analysis between GPI-high and GPI-low groups in immunotherapy cohorts. *P < 0.05; ***P < 0.001; ns, not significant.

So far, the correlations among pyroptosis subtypes, GPI, prognosis, and TIME in glioma have been demonstrated. Further studying the potential therapeutic value of pyroptosis is promising, especially in the context of chemoimmunotherapy. **Figure 9A** shows that the C1 had the lowest TMZ IC_50_ among the three pyroptosis subtypes. **Figures 9B, C** show that the GPI-high group had a lower TMZ IC_50_, and the GPI levels were negatively correlated with TMZ IC_50_ (r = -0.360, P < 0.001), indicating that patients with higher GPI were more sensitive to TMZ treatment. To explore the predictive value of GPI for ICB response, we used the “Submap” algorithm. **Figure 9D** shows that patients with high GPIs were more responsive to anti-PD1 therapy (nominal P = 0.024; Bonferroni-corrected P = 0.003). To validate the predictive accuracy of GPI for the response to and efficacy of immunotherapy, we selected several external ICB immunotherapy cohorts. **Figures 9E, F** **s**how that the GPIs of responders were higher than those of non-responders. **Figures 9G, H** show that GPI has high accuracy in predicting patients’ response to ICB (Snyder et al. cohort: GPI AUC = 0.812; Nathanson et al. cohort: GPI AUC = 0.841), and GSDMD may be one of the potential molecules influencing patient responses to immunotherapy (Snyder et al. cohort: GSDMD AUC = 0.812; Nathanson et al. cohort: GSDMD AUC = 0.909). **Figures 9I–L** show that the GPI-H group had a longer survival time than the GPI-L group. These results indicate that the high GPI may be associated with improved response to and efficacy of ICB therapy

### Potential Small-Molecule Compounds Based on GPI

**Figure 10:**
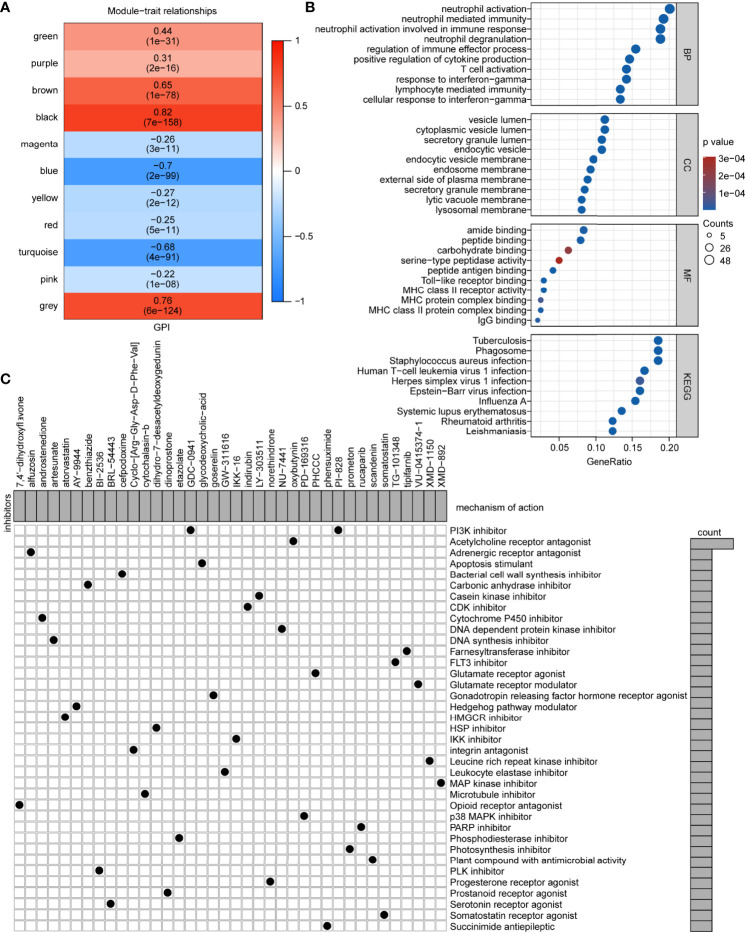
Potential small-molecule compounds based on GPI. **(A)** Module-trait relationships from the WGCNA. **(B)** Bubble map of GO functional and KEGG pathway enrichment analyses. BP, biological process; CC, cellular component; MF, molecular function. **(C)** MOA analysis results using the CMap database showing small-molecule compounds with corresponding mechanisms of action.

To determine how pyroptosis can be activated or suppressed in tumor cells, we explored potential small molecules based on GPI. First, WGCNA was performed and 10 co-expression gene modules based on a soft threshold (power) of 11 (**Figures S10A, B**; namely, green, purple, brown, black, magenta, blue, yellow, red, turquoise, and pink, where gray module is considered a collection of genes that cannot be assigned to any module) were obtained (**Figure S10C** and **Table S9**). Among the 10 modules, brown (r = 0.65, P < 0.05), black (r = 0.82, P < 0.05), blue (r = -0.70, P < 0.05), and turquoise (r = -0.68, P < 0.05) were associated with GPI (**Figure 10A**). To further identify a module that correlated with immunity, we analyzed the main functions of these four GPI-related modules one by one in the STRING database and identified that the brown module genes were mainly related to immunity. We identified 767 upregulated and 973 downregulated DEGs between the GPI-H and GPI-L groups *via* DEG analysis (**Figure S10D** and **Table S10**). The Venn diagram (**Figure S10E**) shows 249 upregulated genes and the only downregulated gene in the brown module (**Table S11**). The GO functional and KEGG pathway enrichment analyses for these genes showed that biological processes (BP) were mainly enriched in neutrophil activation, neutrophil-mediated immunity, neutrophil activation involved in immune response, neutrophil degranulation, regulation of immune effector process, positive regulation of cytokine production, T cell activation, response to interferon-gamma, lymphocyte-mediated immunity, and cellular response to interferon-gamma. The cellular components (CCs) were mainly enriched in the vesicle lumen, endocytic vesicle, endosome membrane, secretory granule membrane, lysosomal membrane, among other processes. The molecular functions (MF) were mainly enriched in peptide antigen binding, Toll-like receptor binding, MHC class II receptor activity, MHC protein complex binding, and MHC class II protein complex binding. The KEGG pathways were mainly enriched in tuberculosis, phagosome, and human T cell leukemia virus 1 infection. (**Figure 10B** and **Table S12**). Finally, candidate small-molecule drugs and their mechanisms of action were predicted using the CMap database and MOA analysis based on the 249 upregulated genes in the brown module and the top 51 downregulated DEGs (**Table S13**). The results are summarized in **Table S14**, and the top 38 potential small-molecule compounds and their corresponding mechanisms of action are shown in **Figure 10C**. These results provide new insights into the mechanisms of triggering or inhibiting pyroptosis *via* drugs in glioma.

### GSDMD Is Associated With Prognosis and Anticancer Immunity Pan-Cancers

Building on the previous analysis for the correlation of GPI with glioma pyroptosis subtypes, prognosis, and TIME features, we focused on GSDMD to provide further insights into future anticancer research (a complete list of cancer-type abbreviations is provided in **Table S15**). **Figure S11A** shows that the expression of GSDMD was significantly upregulated in 14 cancer types: GBM, LGG, UCEC, BRCA, KIRP, KIPAN, HNSC, KIRC, LIHC, SKCM, BLCA, PAAD, TGCT, and CHOL, while it was downregulated in 15 cancer types: LUAD, ESCA, STES, COAD, PRAD, STAD, LUSC, WT, THCA, OV, UCS, ALL, PCPG, ACC, and KICH. **Figure S11B** shows that in five cancer types (LGG, KIPAN, GBM, UVM, and ACC), high expression of GSDMD was associated with poor prognosis, while in three cancer types (KIRP, SKCM-M, and SKCM), its low expression correlated with poor prognosis. **Figure S12** and **Table S16** show that the expression of GSDMD was positively correlated with the majority of immunomodulators in OV, LGG, BLCA, LUSC, UVM, HNSC, KIPAN, STES, STAD, COAD, PRAD, SARC, PCPG, TGCT, KIRC, GBM, SKCM, LUAD, KICH, ESCA, CESC, THCA, and LAML and negatively correlated with those of THYM. **Figure S13** and **Table S17** show that GSDMD correlates positively with immune scores in GBM, LGG, CESC, LUAD, COAD, BRCA, ESCA, STES, SARC, KIPAN, STAD, PRAD, UCEC, HNSC, KIRC, LUSC, THYM, LIHC, MESO, SKCM-M, SKCM, OV, TGCT, PCPG, SKCM-P, UVM, UCS, BLCA, and KICH, whereas it was not negatively correlated with those of any cancer type. **Figure S14** and **Table S18** show that GSDMD expression was positively related to MHC score, EC score, and IPS in most cancers but negatively correlated with SC and CP scores, implying that GSDMC and GSDMD expression is correlated with immunogenicity in many cancers.

## Discussion

We systematically analyzed the transcriptional and genetic heterogeneity of pyroptosis executors, identified three pyroptosis subtypes, constructed a pyroptosis-related scoring system, and described the effects of pyroptosis on glioma in multiple dimensions. Here, we provide valuable information about the potential interrelationships among pyroptosis subtypes, GPI, clinical features, molecular characteristics, the immune microenvironment, and the immunotherapeutic response in glioma patients. Based on these interrelationships, our research may contribute to the development of appropriate novel therapeutic strategies for glioma.

Our study shows that GSDMC, GSDMD, and PJVK have transcriptional heterogeneity and are associated with glioma prognosis. Studies have demonstrated that GSDMC is highly expressed in metastatic melanoma (53) and that the knockdown of GSDMC inhibits the proliferation of colorectal cancer cells (54), while the expression of GSDMC is suppressed in esophageal and gastric cancers (13). Our results indicate that the expression of GSDMC decreased as the tumor grade increased and is a factor that indicates favorable prognosis. Thus, it is unclear whether GSDMC promotes or inhibits cancer development. GSDMD, one of the most important executors of pyroptosis, is widely expressed in various human tissues (10, 55). A previous study showed that GSDMD expression was negatively correlated with OS and increased after TMZ treatment in a time-dependent manner in glioma (20), which is consistent with our results. These results imply that GSDMD could be a novel prognostic biomarker as well as a marker of sensitivity to TMZ in glioma. Previous studies have demonstrated that all known mutations in PJVK are associated with deafness (56–58), but few studies have shown a link between PJVK and cancer. Here, we found that high PJVK expression correlates with favorable OS, indicating that further exploration of the role of PJVK in cancer development and treatment has broad prospects. Although GSDMA, GSDMB, and GSDME expression was not significantly correlated with glioma prognosis, it had significantly different levels in different clinical or molecular subtypes, suggesting that their potential value in glioma remains to be investigated. In conclusion, although all gasdermins may act as executors of pyroptosis in glioma, they play different roles and have different effects, possibly having opposite effects. The absence of a clear correlation between gasdermin expression and glioma prognosis likely reflects the complex role of pyroptosis in tumorigenesis. Thus, it may be better to assess the pyroptosis status than to explore individual executors.

Based on the pyroptosis-related genes, we defined three pyroptosis subtypes with significant differences in the clinical and TIME characteristics of glioma. Furthermore, we developed a GPI associated with prognosis and the infiltration of antitumor immune cells in glioma. Our study indicates that the pyroptosis subtype C1 is characterized by high GPI, while the subtype C2 and C3 are characterized by low GPI. Considering the expression of GSDMD—the primary executor of pyroptosis—the glioma pyroptosis subtype C1 and a high GPI may represent a potentially activated status of pyroptosis, while the glioma pyroptosis subtype C2 and C3 and a low GPI may represent a potentially suppressed status of pyroptosis. The pyroptosis subtype C1 and a high GPI were found to be associated with glioma progression and a worse prognosis, whereas the subtype C3 and a low GPI were found to be associated with glioma suppression and a better prognosis. These findings imply that although activating pyroptosis leads to cell death, it still promotes glioma malignancy. However, patients with high GPIs were also found to be more sensitive to TMZ and anti-PD1 therapy than those with low GPIs. These paradoxical results can be interpreted from several perspectives. On the one hand, pyroptosis, a lytic and proinflammatory type of regulated cell death, is characterized by cell swelling, lysis, and the release of numerous proinflammatory factors, including IL-18, ATP, IL-1β, and HMGB1, which can promote tumor growth and progression (59–65). Chronic inflammation can increase the risk of cancers through multiple mechanisms involving not only the tumor but also tumor-infiltrating stromal cells and immune cells (66). On the other hand, the TME is composed of interstitial fluid, the extracellular matrix, and other components (tumor cells, immune cells, and stromal cells) (67), and the balance between tumor-promoting and tumor-suppressing factors in the TME regulates tumor growth (68). Therapy-induced acute inflammation boosts antitumor immunity by promoting antigen-presentation by recruiting immune cells (such as mature dendritic cells and macrophages) to the TME (11). Induction of tumor cell pyroptosis can create an opportunity to reverse the immune desert phenotype, turning a “cold” tumor into a “hot” tumor (11, 69, 70).

In this study, we found that gasdermins were significantly correlated with the biomarkers of GASCs, GAICs, and GSCs. The stromal score and immune score, calculated to predict the overall level of infiltrating stromal and immune cells, respectively, were both significantly increased in the pyroptosis subtype C1 and positively correlated with GPI. Specifically, for GASCs, the infiltration of astrocytes, endothelial cells, fibroblasts, lymphatic endothelial cells, mesenchymal stem cells, microvascular endothelial cells, and skeletal muscle cells significantly increased in C1 and was positively correlated with GPI. For GAICs, the infiltration of memory B cells, CD8^+^ T cells, resting memory CD4^+^ T cells, activated memory CD4^+^ T cells, regulatory T cells, resting NK cells, M0 macrophages, M1 macrophages, M2 macrophages, activated myeloid dendritic cells, activated mast cells, and neutrophils significantly increased in C1 and positively correlated with GPI. In addition, mRNAsi decreased in C1 and was negatively correlated with GPI. GASCs significantly enhance the proliferation and tumorigenicity of GSCs (71, 72) and promote glioma angiogenesis and growth *in vitro* and *in vivo* (73, 74). Multiple studies have shown that the infiltration of immune-suppressing cells (including lymphocytic B cells, M2 macrophages, myeloid dendritic cells, and regulatory T cells) enhances tumor growth and progression (75–80). A previous study demonstrated that high mRNAsi was present in GBM rather than in LGG and was associated with a poor prognosis, which is consistent with our results (32). Thus, the activation of pyroptosis may lead to an inhibitory immune microenvironment and affect the characteristics of GSCs, thereby promoting tumor progression, which could explain why subtype C1 and high GPI values were associated with aggressive phenotypes of glioma. However, studies have demonstrated that certain chemotherapeutic drugs, such as cisplatin and paclitaxel, effectively suppress tumor growth and metastasis by evoking the conversion from caspase 3-dependent apoptosis to pyroptosis (17, 81–83), which could explain why high activating levels of pyroptosis are associated with high TMZ sensitivity in glioma. Of note, Wang et al. showed that pyroptosis-inducible therapy increased the infiltration of CD8^+^ cells, CD4^+^ T cells, and NK cells in mammary tumor grafts, and the pyroptosis of less than 15% of tumor cells was sufficient to clear all 4T1 experimental breast tumors (84). Furthermore, pyroptosis in 4T1 tumor cells induced the polarization of M1 macrophages (84). Zhang et al. also found that in the pyroptosis-activated TIME, CD8^+^ T and NK cells induced tumor cell pyroptosis through granzyme B, thus forming a positive feedback loop (18). Similarly, NK cells and CD8^+^ T cells have been recently shown to trigger tumor clearance *via* the GSDMB-granzyme A axis (12), and higher enrichment of NK and CD8^+^ T cells usually reflects better ICB efficacy (85). GSDMD is required for the antitumor function of CD8^+^ T cells (86), and GSDMD deficiency decreases the cytolytic capacity of CD8^+^ T cells (87). Thus, chemotherapy and immunotherapy may rapidly induce pyroptosis and boost antitumor immunity by increasing the recruitment and activation of CD8^+^ and NK cells in glioma, which could explain why the pyroptosis subtype C1 and high GPIs were associated with higher TMZ sensitivity and better anti-PD1 therapy response. Furthermore, we found that gasdermins were correlated with most of the immune checkpoints; the PD-L1 expression and TMB score were increased in C1 and positively correlated with GPI, while the MSI score showed the opposite trend. TMB and MSI scores and PD-L1 levels are important predictive biomarkers for ICB effectiveness (31, 88, 89), which help illustrate the potential predictive ability of GPI for ICB response. In addition, our results show significant differences in genetic alterations in tumor driver genes (*IDH1, PTEN, TP53*, and *ATRX*) among the pyroptosis subtypes C1, C2, and C3, and many cancer-promoting pathways and immune-related processes were greatly enriched in the GPI-H group. Whether the tumor-promoting or tumor-suppressing roles dominate, the role of pyroptosis likely depends on the specific genetic and epigenetic characteristics of the tumor, combined with differences in host inflammatory status and immunity (11). These results indicate that genetic features act as intrinsic factors, leading to differences in pyroptosis status *via* certain pathways, further remodeling the TIME to influence prognosis and therapy effectiveness in glioma. Further studies are required to verify this hypothesis.

This study has some limitations. This retrospective study used publicly available data and algorithms. We collected glioma samples for further analysis and verification. Overall, we explored the function of pyroptosis and developed a pyroptosis-related index for glioma; however, there is a need to determine the mechanisms of certain oncogenes involved, on which we are conducting further research. The pan-cancer analysis suggests that GSDMD plays a potential role in immunotherapy. However, the specific mechanism of action requires further investigation. We hope that the pan-cancer exploration in our study will encourage further studies on this subject.

## Conclusions

Taken together, the glioma pyroptosis subtype C1 and a high GPI were associated with malignant characteristics but may improve the sensitivity to and efficacy of TMZ and ICB treatment, highlighting the importance of a combination of pyroptosis-targeted therapy with chemotherapy and/or immunotherapy for glioma. Thus, pyroptosis of glioma cells may perform the dual function of tumorigenesis and antitumor immunity.

## Data Availability Statement

Publicly available datasets were analyzed in this study. Those data can be found here: https://portal.gdc.cancer.gov/, http://www.cgga.org.cn/index.jsp, 924 https://www.ncbi.nlm.nih.gov/geo/query/acc.cgi?acc=GSE43378, https://portals.broadinstitute.org/ccle/about, and https://xenabrowser.net/.

## Author Contributions

Research design: HW, XZ, YS. Data analysis: YC, KL. Manuscript writing: YC, KL. Manuscript revision: YC, KL, JL, XL, WX, ZZ, SX, YZ, PC, YM, ZS, LH, HW, XZ, YS. All authors have read and approved the final manuscript.

## Funding

The study was supported by the Science and Technology Program of Guangzhou, China (No. 201903010048); National Nature Science Fund of China (No. 81872064); the Natural Science Fund of Guangdong Province, China (No. 2020A1515010122 and 2021A1515012465). The funders had no role in study design, data collection, data analysis, decision to publish, or preparation of the manuscript.

## Conflict of Interest

The authors declare that the research was conducted in the absence of any commercial or financial relationships that could be construed as a potential conflict of interest.

## Publisher’s Note

All claims expressed in this article are solely those of the authors and do not necessarily represent those of their affiliated organizations, or those of the publisher, the editors and the reviewers. Any product that may be evaluated in this article, or claim that may be made by its manufacturer, is not guaranteed or endorsed by the publisher.
